# Phase 2 Study Results of Oral ALZ‐801 (Valiltramiprosate) in Early Alzheimer’s Disease Shows Potential Disease Modification Effect: 2‐Year Effect on Plasma Biomarkers, Volumetric MRI, Cognition Outcomes and Clinical Benefit Analysis

**DOI:** 10.1002/alz.092950

**Published:** 2025-01-09

**Authors:** John A. Hey, Susan Abushakra, Kaj Blennow, Patrick Kesslak, Jakub Hort, Niels D. Prins, Katerina Sheardova, Jean Schaefer, Adem Albayrak, Winnie Pak, Aidan Power, Martin Tolar

**Affiliations:** ^1^ Alzheon, Inc., Framingham, MA USA; ^2^ University of Gothenburg, Gothenburg Sweden; ^3^ Alzheon Inc., Framingham, MA USA; ^4^ Memory Clinic, Department of Neurology, Charles University, Second Faculty of Medicine and Motol University Hospital, Prague Czech Republic; ^5^ Brain Research Center, Amsterdam Netherlands; ^6^ International Clinical Research Center, St. Anne’s University Hospital, Brno Czech Republic; ^7^ Alzheon, Framingham, MA USA

## Abstract

**Background:**

ALZ‐801 (valiltramiprosate), an oral brain‐penetrant amyloid‐oligomer inhibitor in Phase 3 testing in APOE4/4 homozygotes (APOLLOE4 trial). A 2‐year Phase 2 biomarker study was completed evaluating ALZ‐801 (265 mg BID) on plasma biomarkers, MRI, cognition, and clinical benefit in EAD APOE4 carriers. At trial end, subjects could enroll in a 1‐year long‐term extension with an ongoing biomarker and cognition analysis.

**Method:**

This 104‐week, open‐label study enrolled 84 subjects (MMSE 22‐30, CDR‐G 0.5‐1, positive amyloid‐PET or CSF biomarkers). Plasma and clinical testing occurred every 13 weeks; MRI every 52 weeks. Primary outcome was plasma p‐tau_181_; cognitive tests were Rey Auditory‐Verbal‐Learning Test (RAVLT) and Digit‐Symbol‐Substitution Test (DSST). Dr. Blennow’s Laboratory (Sweden) conducted plasma biomarker analyses (Simoa, Euroimmun assays). Hippocampal volume and cognitive tests were compared to matched group from ADNI‐1 observational study using simple matching (age, APOE4 genotype, disease stage) and propensity scores as covariates in the MMRM analysis. Clinical and HV benefit was determined by Cohen’s d score. For plasma biomarkers, observed data changes‐from‐baseline were analyzed with 2‐sided simple t‐tests.

**Result:**

84 subjects were enrolled; 51% female, 69 years, MMSE 26.0, 70%/30% had MCI/Mild AD; 70 completed 104‐weeks. Plasma p‐tau_181_ showed significant reductions at all timepoints reaching 31%‐43% over 52‐104 weeks (p = 0.045); Aβ42 decreased ∼4% over 104 weeks (p = 0.042). Hippocampal atrophy (3.6%) was ∼28% lower than matched external control (ADNI‐1). RAVLT‐total memory and DSST remained above/at baseline through 104 weeks; Cohen’s d clinical benefit effect in RAVLT and hippocampal volume ranged from 0.5 to 0.7. Cognitive stabilization correlated with decreased hippocampal atrophy (Spearman’s r = 0.38‐0.43, p≤0.002); and cortical thinning (r = 0.35‐0.58, p≤0.004). Most common TEAE was mild nausea, and no ARIA‐E was observed.

**Conclusion:**

Over 2 years, oral ALZ‐801 reduced plasma p‐tau_181_. Cognitive stabilization correlated with reduced brain atrophy, showing treatment benefit compared to external controls. Cohen’s d values for hippocampal volume and cognition suggest strong clinical benefit. No ARIA‐E/vasogenic edema was detected. These biomarker results support the disease‐modifying effects of ALZ‐801 in Early AD with promising clinical efficacy and favorable safety in APOE4 carriers. A Phase 3 78‐week trial in APOE4/4 homozygotes Early AD (EAD) is ongoing with results expected in 2024.

